# New metallo β-lactamase NDM-1

**Published:** 2010-11

**Authors:** D. Raghunath

**Affiliations:** Sir Dorabji Tata Centre for Research in Tropical Diseases, Innovation Centre Indian Institute of Science Campus Bangalore 560 012, India sdtc265iisc@gmail.com

Antimicrobial resistance has been recognized since the beginning of the antibiotic era in mid 20^th^century. The availability of newer agents minimized the clinical threats, however, depleting reserves and “the relentless and Dizzying Rise of antimicrobial Resistance”^1^ have raised the spectre of a post-antibiotic scene with untreatable infections. Bacteria have countered the introduction of successive classes of antibiotics by developing a variety of resistance mechanisms. The versatility of the bacterial genomic pool and our incomplete knowledge of the modifying process have been demonstrated^2^. The history of β-lactamases starting from the discovery of the TEM in 1965 has demonstrated how the microbial world keeps pace with technical advances^3^. At the top of the pyramid are the carbapenemases. The metallo β-lactamases (MBL) are the more versatile enzymes that can convert the host bacteria into almost total β-lactam insusceptibily^4^. Infections by such organisms are practically untreatable by β-lactam antibiotics, which are the most favoured agents used in Gram negative sepsis.

β-lactamases capable of hydrolysing carbapenems are either serine enzymes or metallo -β-lactamases that have a divalent cation (usually zinc) regulating the active site. The first bacterial isolate carrying the MBL later designated *bla*_NDM-1_ was a *Klebsiella pneumoniae* urinary isolate from a patient with insignificant bacteria along with other multiresistant bacteria from wound swabs. Faecal cultures done to identify the reservoir of infections yielded a strain of *Escherichia coli* carrying a similar resistance factor suggesting the possibility of conjugational transfer in the gut. However, there are differences in the molecular properties in the two bacteria indicating *in vivo* rearrangement. The properties, biological characters and phylogenetic relationship to other MBLs have been reported^5^. Briefly, *bla*_NDM-1_ is a novel MBL with ability to confer resistance to almost all the β lactams. The NDM-1 gene is normally carried on a variety of plasmids along with other resistance factors. The bacteria also showed broad antibiotic resistance to all classes except colistin and ciprofloxacin. Rarely the gene was integrated into the chromosome. The sequencing of the gene showed that it was a new enzyme unrelated to hitherto known MBLs. The closest known type was VIM-1/VIM-2 sharing a 32.4 per cent identity. The NDM-1 has an unique additional sequence. The properties of the protein, kinetic characteristics *etc* are reported in detail^5^. In summary, NDM1 confers on its host bacteria almost all β lactam resistance and is accompanied by extensive antibiotic resistance. Such bacteria would be difficult to eliminate in clinical situations.

Karthikeyan *et al*^6^ presented the result of a larger study including the epidemiology of the resistance mechanism. Two sites at Chennai and Haryana covering 3521 and 198 isolates respectively showed 1 and 13 per cent prevalence of NDM1 carrying *Enterobacteriaceae*. The Chennai isolates included different species whereas all the Haryana isolates were *K. pneumoniae*. The sources of the isolates were community acquired urinary tract infections, pneumonia and blood stream infections. In the UK starting from 2008, isolates with NDM1 enzymes became the “dominant carbapenemase-producing *Enterobacteriaceae*”. A number of isolates from nine locations in India, eight cities in Pakistan and Dhaka, Bangladesh have been confirmed to carry NDM1 (by PCR)^6^ indicating that the plasmid is perhaps quite widely distributed in the subcontinent. While this fact leads credence to the ‘Indian’ origin, it may not be necessarily so. The widespread prevalence of multidrug resistant *Salmonella* Typhimurium DT104 in India was traced to trans border induction and rapid dissemination thereafter^7^. Likewise, the now widely prevalent penicillin resistant *Streptococcus pneumoniae* was from Spain^8^. However, the statement *bla*_NDM-1_is widespread in the Indian environment is true. In fact, National Institute of Cholera and Enteric Diseases (NICED), Koltaka, has demonstrated the plasmid in nine surveillance rectal isolates and four cases of neonatal sepsis from a remote area (Birbhum District) [personal communication : Sulagna Basu (NICED) and Arun Singh (Institute of Post Graduate Medical Educaction & Research and SSKM Hospital, Kolkata)]. Our experience of a fulminant fatal multiple antibiotic resistant *Pseudomonas aeruginosa* speticaemia in a 34 yr old woman being prepared for bone marrow transplant, brings the issue to the fore^9^. Septicaemia caused by a bacterium carrying NDM-1 would be well nigh impossible to treat if it has the accompanying resistances. However, the mere presence of the organism in the environment would not translate to infection if control measures are in position, as they would be, in the major corporate hospitals that cater to medical tourism.

The study of antibiotic resistance, a major field of scientific endeavour is hardly noticed by lay media. However, the paper by Karthikeyan *et al*^6^ has generated heated discussion in professional circles as well as the print and electronic media in India. Two aspects of the publication have provoked the discussions. One has been the designation of the new enzyme as NDM-1 ascribing the origin of the resistance factors to New Delhi, the capital of India. Essentially, this is in line with the current practice of naming carbapenemases by adopting the initials of the city/locality of origin of the first isolate carrying the enzyme, *e.g.* SPM-1 has been named after Sao Paulo^10^. In the present case, the enzyme was first detected in Sweden in a urinary isolate. The *K. pneumoniae* strain carrying the enzyme was colonizing the patient’s urinary tract. The patient had travelled from New Delhi, hence, the designation^5^. The patient’s travel history and prior hospitalization within India raises the possibility of the organism having been acquired outside New Delhi. Nevertheless, the designation as NDM-1 is not really objectionable but questionable as the origin was not proven conclusively. The second aspect that has generated a heated debate in Indian professional and media circles is the assertion of the authors that patients from the West who would travel to the Indian sub-continent for elective surgery are at risk of post-operative infections due to bacteria carrying NDM-1 resulting in higher treatment costs. This would negate any economy that would accrue to the NHS from contracted outsourcing of elective surgery to “countries such as India”^11^. Kartikeyan *et al*^6^ mention about 17 of 29 UK patients yielding isolates with NDM-1 having travelled to India or Pakistan within a year and that 14 of them had been admitted to hospitals in the region. However, except in two pairs, the *K. pneumoniae* and *E. coli* isolates were unrelated to each others. Making a putative policy modifying statement based on the evidence points to suscipicous non-scientific agenda. Strangely, the National (UK) Resistance Alert in vol 3 (4) of the Health Protection Report (referred to 28)^12^ in this paper warns about carbapenemases but does not mention about the MBL *bla*_NDM-1_. However, a fresh alert specifically mentioning *bla*_NDM-1_ has been issued on July 3, 2009^12^. It is seen from the Alerts that importation of carbapenemase (other than NDM-1) carrying organisms occurs from a number of sites in the Eastern Mediterranean too. In fact, the initial paper describing NDM-1^5^ with the same corresponding author hints at such a preconception. This should have been addressed during peer review of the *Lancet Infections Disease* paper. Notably, ‘medical tourism’ is a rapidly growing field in India and is attracting a number of patients from the West. The private medical care ‘industry’ in India is looking forward to significant growth in the near future^13^. Any adverse reports on the safety of such patients who came to India for elective surgery without substantive evidence is likely to impact hospitals catering to such patients in India.

Curiously, there has been little evidence of clonal spread of NDM-1 bearing *E.coli* or *K.pneumoniae* except in the case of the isolates within Haryana. What is more, the epidemiology of the focus has not been systematically studied either. This needs to be done soon. By and large, most of the other reports including the study by Deshpande *et al*^14^ are based on PCR detections. It is hoped some of these locales, particularly in the better institutions, will be studied in greater depth. In fact, if hospital infection control mechanisms are in position these isolations would already have activated investigations. The availability of a national “Antibiotic Resistance Monitoring and Reference Laboratory” would have facilitated the molecular investigations.

The disorganized state of antibiotic therapy in India has been well recognized. The prescription habits, lack of infection control and easy access to even top line antibiotics add up to a dismal scenario prompting observers to write an ‘obituary’ to antibiotic treatment^15^. This is probably the view from a big corporate tertiary hospital that attracts ‘failed’ cases. Nevertheless, these factors along with poor sanitation and personal hygiene foster the generation of resistant bacteria and their dissemination. Even in the case of organism carrying the NDM-1 plasmid a significant proportion of isolates was from patients infected in the community where one would not expect a heavy antibiotic selection pressure. In this field, as in others, China is a competitor. The alarm that has been raised in the case of China^16^ has an echo in India.

The belief that multiresistant pathogens are physically hampered and incapable of surviving in nature is no longer valid. Numerous instances of hospital generated or strains acclimatized to high levels of environmental antibiotic pressures living and spreading ‘merrily’ in the community are now known. Therefore, the incapacity of the weaker sections of our population to procure costilier antibiotics no longer protects them from multiresistant organisms^17^. It is thus, not surprising that NDM-1 bearing *Enterobacteriaceae* have been seen to be present in the community. The global multiresistance scene has been summarized by Cars^18^. Of course, it has long been realized that human usage is only one of the determinants of the environmental pressure driving the evolution of antibiotic resistance. Usage in animal husbandry and run off from production facilities are two other major components that need to be addressed. The Indian environment has plenty of these. Industrial effluent management is still developing. Could such factors be at play in the genesis of the Haryana clone which has distinct properties^6^?

Much as our national sentiments are tickled by the NDM-1 report, the development is alarming. The case vignette in the editorial^9^ is not unusual. The tragedy is replicated frequently. It is high time that we put in place practices and institutions that regulate antibiotic therapy. Essentially, the practices should aim at diminishing environmental antibiotic levels and inhibiting the spread of resistance factors.

A serious shortcoming is the absence of an Indian National Reference Laboratory for following antibiotic resistance trends and an institution for formulating and for directing antibiotic policies. Smaller (advanced) countries have such facilities, but, a large diverse nation - India does not. If such an institution was available, Indian workers like Karthikeyan need not have collaborated with others countries and subjected themselves to the embarrassment of disassociating (even under pressure) with aspects of a consensual publication^19^. The way forward has been indicated elsewhere^9^. We need to act fast as “The window of opportunity is rapidly closing”^18^.
